# Arg156 in the AP2-Domain Exhibits the Highest Binding Activity among the 20 Individuals to the GCC Box in BnaERF-B3-hy15, a Mutant ERF Transcription Factor from *Brassica napus*

**DOI:** 10.3389/fpls.2016.01603

**Published:** 2016-10-27

**Authors:** Jing Zhuang, Meng-Yao Li, Bei Wu, Yan-Jun Liu, Ai-Sheng Xiong

**Affiliations:** State Key Laboratory of Crop Genetics and Germplasm Enhancement, College of Horticulture, Nanjing Agricultural UniversityNanjing, China

**Keywords:** arginine, *Brassica napus* L., ERF factor, GCC element, site-directed mutagenesis, yeast-one hybrid

## Abstract

To develop mutants of the ERF factor with more binding activities to the GCC box, we performed *in vitro* directed evolution by using DNA shuffling and screened mutants through yeast one-hybrid assay. Here, a series of mutants were obtained and used to reveal key amino acids that induce changes in the DNA binding activity of the BnaERF-B3 protein. With the BnaERF-B3-hy15 as the template, we produced 12 mutants which host individual mutation of potential key residues. We found that amino acid 156 is the key site, and the other 18 mutants host the 18 corresponding individual amino acid residues at site 156. Among the 20 individuals comprising WT (Gly156), Mu3 (Arg156), and 18 mutants with other 18 amino acid residues, Arg156 in the AP2-domain is the amino acid residue with the highest binding activity to the GCC box. The structure of the α-helix in the AP2-domain affects the binding activity. Other residues within AP2-domain modulated binding activity of ERF protein, suggesting that these positions are important for binding activity. Comparison of the mutant and wild-type transcription factors revealed the relationship of protein function and sequence modification. Our result provides a potential useful resource for understanding the trans-activation of ERF proteins.

## Introduction

Plants are affected by various abiotic stresses during their life cycle. High salt, drought, and extreme temperature are major factors limiting plant growth and development, and considerably affect the efficiency of agricultural production. When plants are exposed to adverse stress, a complex signal transduction network will mediate resistance to the stress (Seki et al., 2001; Shinozaki et al., 2003; Gutterson and Reuber, 2004). Numerous transcription factors have been identified and associated with adaptive strategies of plants; these transcription factors, including those in the AP2/ERF family, bind to the DNA-binding elements at the promoter of downstream target genes and regulate gene expression (Yamasaki et al., 2013; Franco-Zorrilla et al., 2014). The AP2/ERF transcription factor family is one of the families involved in plant development, signal transduction, and stress response; this family can be further divided into four subfamilies: AP2, RAV, DREB, and ERF (Sakuma et al., 2002; Zhuang et al., 2008).

The two largest subfamilies DREB and ERF present a high similarity in their AP2-domains, but the core amino acids determine their binding activity to different *cis* elements (Yamaguchi-Shinozaki and Shinozaki, 1994; Fujimoto et al., 2000). Most DREB proteins bind to the DRE element, whereas the ERF subfamily factors mainly recognize the GCC box (Sakuma et al., 2002; Liu et al., 2006). The DRE element with the core sequence AGCCGAC was found to be important to the DNA-binding specificity of DREB proteins, with three positions (C4, G5, and C7) as key bases for binding affinity (Sakuma et al., 2002). In the AP2-domain of two *Arabidopsis* DREB proteins, Val14 and Glu19 are important to DNA-binding specificity; in particular, Val14 is critical for determining DREB-binding efficiency (Sakuma et al., 2002). Similar results were also found in the maize DREB protein (Qin et al., 2003). A GCC box has been found in the promoter region of ethylene-inducible genes, Hao and his colleagues reported that the 1st G, the 4th G, and the 6th C are essential in the GCC box for specific binding to the respective domain of ERF transcription factors (Hao et al., 1998). Some DREB and ERF factors were reported to bind to both GCC and DRE elements, such as CBF1-F (Hao et al., 2002), BnDREBIII-1 (Liu et al., 2006), and Tsi1 (Park et al., 2001).

In our previous studies, we isolated the gene *BnaERF-B3-hy15* encoding an ERF transcription factor from *Brassica napus* L. cv. Huyou15 and obtained three BnaERF-B3 mutants by DNA shuffling (Jin et al., 2010a). The yeast one-hybrid system showed that the three modified proteins exhibit strong binding activity to the GCC box, but the original protein showed almost no binding activity. The overexpressed mutant *BnaERF-B3-mu3*, which shows a high binding activity to the GCC box, exhibits a higher freezing tolerance than the original *BnaERF-B3* in transgenic *Arabidopsis* (Xiong et al., 2013). Sequence alignment results revealed that the 12 amino acid residues differ between BnaERF-B3-hy15 and the three mutants, but only Gly156 is located in the AP2-domain.

Among the 12 mutant amino acids, the amino acid that shows a high binding activity to the GCC box, is it located in the AP2-domain or in the peripheral area? Site-directed mutagenesis was repeatedly performed to identify key sites in protein sequences for functional analysis (Lu et al., 2002; Nguyen et al., 2012; Guan et al., 2014). Previous molecular analyses of DREB or ERF mutants through site-directed mutagenesis provided insights into the amino acid sequences in the AP2-domain that are decisive for protein functions (Zhao et al., 2007; Sun et al., 2008). To identify the key site (s) which determined the DNA binding activity to the GCC box, we established a series of mutants of BnaERF-B3-hy15 through site-directed mutagenesis and screened mutants through yeast one-hybrid assay. Our results revealed that Arg156 in the AP2-domain is the amino acid residue with the highest binding activity to the GCC box in the mutant and wild type BnaERF-B3. This work can be used for further research on ERF transcription factor function.

## Materials and methods

### Plant materials and gene cloning

The rapeseed cultivar (*B*. *napus*. L. cv. Huyou15) was used as experiment material in this study. Plants were grown in pots containing soil, vermiculite, and peat moss (1:1:1 by volume) in a growth chamber at 22°C for 20 d under a 16 h day/8 h night cycle. The *BnaERF-B3-hy15* gene encoding the AP2/ERF protein was cloned from *B*. *napus* L. Huyou15 as previously described, and three mutants were obtained by Jin et al. (2010a). Two-month-old plants of *A. thaliana* (L.) Heynh. (ecotype columbia) were used to clone *AtCBF* genes.

### Site-directed mutagenesis of the *BnaERF-B3-hy15* gene

**Figure 1:**
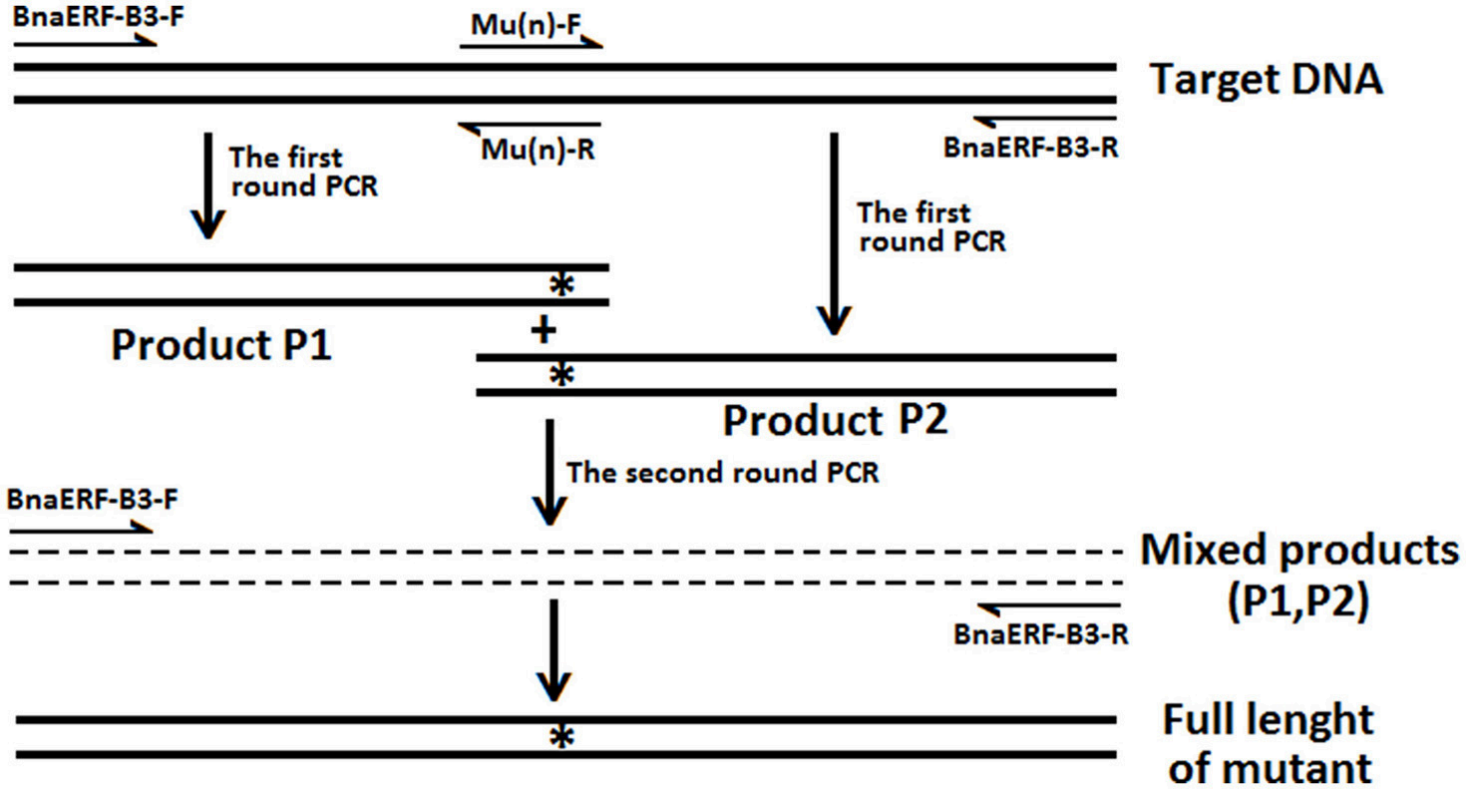
**The strategies and primers of site-directed mutagenesis of BnaERF-B3**. “n” Represent different mutants. “^*^” Represent the mutation sites.

The details of the site-directed mutagenesis of the *BnaERF-B3-hy15* and *CBF* genes to generate mutant genes were accorded to the overlap extension PCR strategy (Xiong et al., 2004, 2006; Peng et al., 2006). The strategy for site-directed mutagenesis is shown in Figure 1. Briefly, the upstream product (Product P1) and downstream product (Product P2) were amplified by first-round PCR with two pairs of oligonucleotides, respectively. Then, the full-length mutant fragment was amplified using a second round PCR with outer pair primers. All the mutagenesis products were ligated to the pMD-19 simple-T vector (TaKaRa, Dalian, China) for DNA sequencing identification and enzyme digestion. All the primers used to clone or synthesize mutations were listed in Table S1.

### Construction of yeast expression vectors

The amplification mutagenesis products were inserted between the *Bam* HI and *Sac* I sites of the yeast expression vector pPC87, which was reconstructed from pPC86. The vector of pPC87 hosts the Trp synthesize gene and contains a galactose-inducible (GAL4) protein activating the domain under the control of the yeast alcohol dehydrogenase (ADH1) promoter. A 75-bp fragment containing the GCC element (5′-ACCCTC GAGCGGATAACAATTTCACACAGGGGCGGCTCTTAGGCGGCTCTTATAAGAGCCGCCGGAT CCGGGCCC-3′, GenBank No. AF394909) was inserted into the vector G222, which could synthesize Ura; the vector also contained the reporter gene *LacZ* under the control of the *iso*-1-cytochrome C (CYC1) minimal promoter. This recombinant plasmid was used as a bait plasmid in the yeast one-hybrid system (Zhang Y. et al., 2009; Jin et al., 2010a,b).

### Yeast one-hybrid and β-galactosidase activity assay

The bait plasmid was transformed into the yeast strain EGY48. Subsequently, the pPC87 recombinant plasmids were transferred into yeast cells, which could grow on the medium without Ura. The transformants were cultured on SD media without Ura and Trp at 30°C for 3 d. A colony-lift filter assay was used for qualitative analysis of *trans*-activation activity. After the fusion protein interactions with the GCC element, the *lacZ* gene was induced and positive cells (blue) were detected through enzymatic staining with X-gal as the substrate. The relative β-galactosidase activity of the transformant colonies were examined with the *o*-nitrophenyl-β-d-galactopyranoside (ONPG) (Clontech Laboratories, Inc.).

## Results

### Directed evolution and screening the potential key amino acids involved in DNA binding activity

In our previous study, we cloned a member of the ERF family gene, named *BnaERF-B3-hy15*; the DNA shuffling strategy was used to shuffle the *BnaERF-B3-hy15* gene, and three mutants (Mu1, Mu2, and Mu3) of this gene were obtained from variant mutants. The four proteins (BnaERF-B3, Mu1, Mu2, and Mu3) were investigated using yeast one-hybrid assays, and the binding activity to the GCC box relatively varied (Jin et al., 2010a).

**Figure 2:**
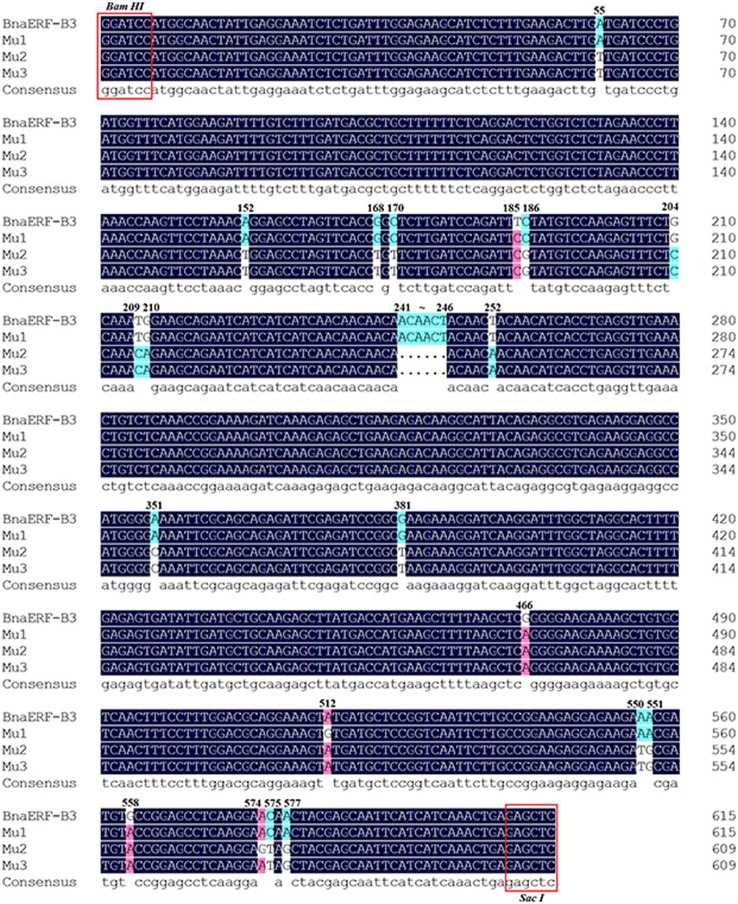
**The mutated nucleic acid sites among the three mutants and wild-type proteins**. The different color codes represent the sequence homology.

The 3D structure of the proteins showed that the AP2-domain consisted of three β-sheets and one α-helix, which functions in DNA binding. Nevertheless, whether the mutant site plays a key role in the binding activity remains unclear. Here, further sequencing analysis of these four genes showed that a total of 26 nucleic acid sites were altered (Figure 2, Table 1), which led to 12 amino acid site substitutions. A subsequent comparison of the amino acid sequences of four proteins showed that these proteins were highly homologous, only one different amino acid (Gly156) was found in the AP2-domain and α-helix, the remaining mutants were located in the periphery of the domain, and one amino acid (site192) differed between Mu2 (Val192) and Mu3 (Ile192) (Figure S1).

**Table 1:** **Mutated nucleic acid sites and deduced amino acid sites among the three mutants and wild-type proteins**.

**Nucleic acid site**	**55**	**152**	**168**	**170**	**185**	**186**	**204**	**209**	**210**	**241**	**242**	**243**	**244**	**245**	**246**	**252**	**351**	**381**	**466**	**512**	**550**	**551**	**558**	**574**	**575**	**577**
BnaERF-B3	A	A	G	C	T	C	G	T	G	A	C	A	A	C	T	T	A	G	G	A	A	A	G	A	C	A
Mu1	A	A	G	C	C	C	G	T	G	A	C	A	A	C	T	T	A	G	A	G	A	A	A	A	C	A
Mu2	T	T	T	T	C	G	C	C	A	–	–	–	–	–	–	A	C	T	A	A	T	G	A	G	T	G
Mu3	T	T	T	T	C	G	C	C	A	–	–	–	–	–	–	A	C	T	A	A	T	G	A	A	T	G
BnaERF-B3	M	Q	/	A	F		/	M		T			T			/	/	/	G	Y	N		/	T		T
Mu1	M	Q	/	A	S		/	M		T			T			/	/	/	R	C	N		/	T		T
Mu2	L	L	/	V	S		/	T		–			–			/	/	/	R	Y	C		/	V		A
Mu3	L	L	/	V	S		/	T		–			–			/	/	/	R	Y	C		/	I		A
Deduced amino acid site	19	51	/	57	62		/	70		78			79			/	/	/	156	171	184		/	192		193

### Characterization of the role of each of 12 amino acid site substitutions in the binding activity of the GCC box through site-directed mutagenesis

**Figure 3:**
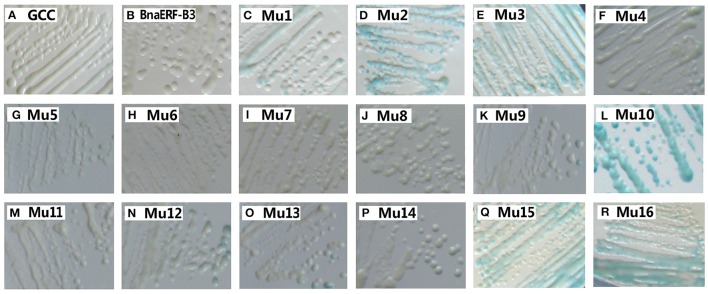
**Yeast-one hybrid analysis of activities of BnaERF-B3 and mutants**. The yeast cells were examined with X-gal after growing on SD/-Ura /-Trp- medium for 2 d at 30°C. GCC: yeast reporter cells carry GCC box (as a control); Mu1–Mu3: the mutants by Jin et al. (2010a). The information of the substitution site and amino acid of all Mu4 to Mu16 were as follows: Mu4: Met19Leu; Mu5: Gln51Leu; Mu6: Ala57Val; Mu7: Phe62Ser; Mu8: Met70Thr; Mu9: Thr78–, Thr79–; Mu10: Gly156Arg; Mu11: Tyr171Cys; Mu12: Asn184Cys; Mu13: Thr192Val, Thr193Ala; Mu14: Thr192Ile, Thr193Ala; Mu15: Gly156Arg, Thr192Val; Mu16: Gly156Arg, Thr192Ile.

**Figure 4:**
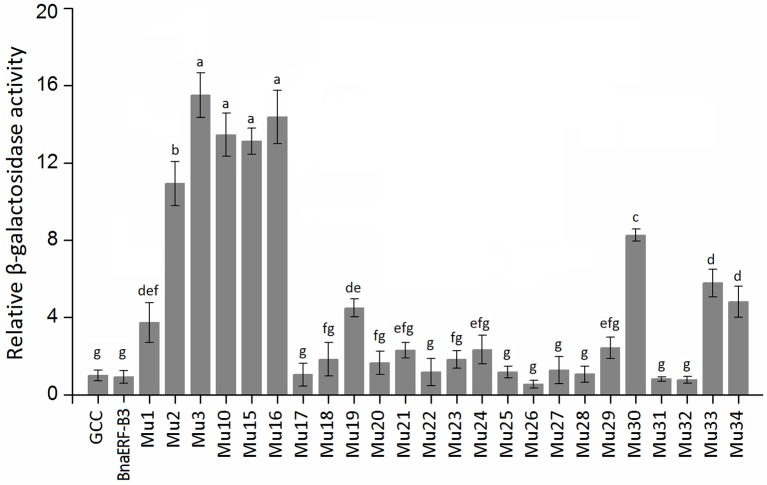
**The relative β-galactosidase activities of BnaERF-B3 and mutants**. Three independent clones were chose to culture in liquid SD/-Ura/-Trp medium for testing the β-galactosidase relative activity with o-nitrophenyl-β-D-galactopyranoside (ONPG) as the substrate. The data are the means ± SD of three replicates. Different letters on the vertical bars indicate significant difference at 0.05 levels.

To identify the amino acid substitution site that may be essential for the binding activity of the mutants, a series of site-directed mutants of BnaERF-B3 were generated according to the sequence alignment results. The strategy for site-directed mutagenesis is shown in Figure 1. All the 11 mutants (Mu4 to Mu14) were confirmed through sequencing, then cloned into the expression vector pPC87, and transformed to yeast, which hosts another vector with the GCC box sequence. Results of the yeast one-hybrid screening showed that the clones of Mu10, where Gly156 was substituted with Arg, appeared dark blue on X-gal-containing plates, but the 10 other mutants (Mu4 to Mu9 and Mu11 to Mu14) did not appear obviously blue (Figure 3). The β-galactosidase activity assay was used to calculate relative activity. As shown in Figure 4, Mu10 showed a 13-fold increase over BnaERF-B3. The differences in the *trans*-activation activity of BnaERF-B3 and the mutants showed that the mutation of Gly156 located in the AP2-domain significantly increased the binding activity. However, the other mutations outside the AP2-domain may have minimal or no influence on the binding activity. Our results indicated that the amino acid residues at site 156 may have an important role in binding to the GCC box.

Only one amino acid differed between Mu2 and Mu3, but the binding activity of these mutants varied. We further designed two sets of site-directed mutagenesis (Thr192Val and Thr192Ile) based on Mu10, which hosts Gly156Arg. Yeast one-hybrid assay and β-galactosidase activity were also used to test the activity of Mu15 (Gly156Arg + Thr192Val) and Mu16 (Gly156Arg + Thr192Ile; Figures 3, 4). Although Mu16 showed a slightly higher increase in activity than Mu15, both mutants exhibited higher activity than BnaERF-B3. This result implied that the mutation of T192 may minimally affect the activity, whereas the mutation of Arg156 played a dominant role.

### Characterization of the role of each altered amino acid in the 156th site in terms of the binding activity to the GCC box through site-directed mutagenesis

The mutation at site 156 from Gly to Arg greatly increased the activity, but we cannot determine whether the activity will be constant even if the 156th amino acid was mutated to the 18 other kinds of amino acid. To investigate the other amino acid residues that affect the binding activity, we replaced the 156th amino acid with the other 18 remaining amino acids into BraERF-B3 and the corresponding mutants were named Mu17 to Mu34.

**Figure 5:**
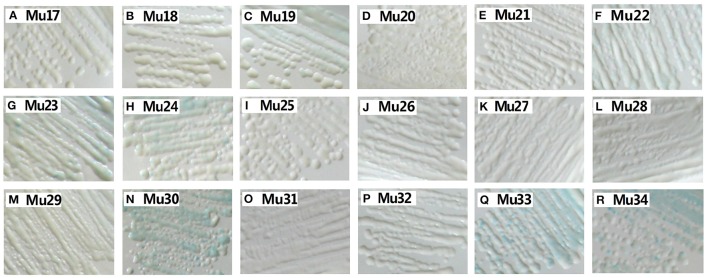
**Yeast-one hybrid analysis of trans-activation activity of mutants of Mu17 to Mu34**. The yeast cells were examined with X-gal after growing on SD/-Ura /-Trp- medium for 2 d at 30°C. The information of the substitution site and amino acid of all Mu17 to Mu34 were as follows: Mu17: Gly156Ala; Mu18: Gly156Val; Mu19: Gly156Leu; Mu20: Gly156Ile; Mu21: Gly156Pro; Mu22: Gly156Phe; Mu23: Gly156Tyr; Mu24: Gly156Trp; Mu25: Gly156Ser; Mu26: Gly156Thr; Mu27: Gly156Cys; Mu28: Gly156Met; Mu29: Gly156Asn; Mu30: Gly156Gln; Mu31: Gly156Asp; Mu32: Gly156Glu; Mu33: Gly156Lys; Mu34: Gly156His.

Yeast one-hybrid analysis results showed that the transformant cells of various mutants had different activity, in which some clones appeared colorless, weakly stained blue, or stained deep blue (Figure 5). The results of quantitative analysis of β-galactosidase activity showed that Mu30 (Gly156Gln) had higher binding ability, followed by Mu33 (Gly156Lys), Mu34 (Gly156His), and Mu19 (Gly156Leu) (Figure 4). However, the activity of all the mutants was lower than that of Mu10 (Gly156Arg). These results illustrated that the space conformation of different amino acids caused conformational changes in the active domain, and the transcription factor showed different levels of activity. Therefore, the 156th amino acid is essential to maintain protein activity. Arg, Gln, Lys, His, and Leu are candidate amino acids for promoting BraEFR-B3 binding to the GCC box; among which, Arg exhibits the highest potential.

### Mutation in the 156th amino acid changes the structure

**Figure 6:**
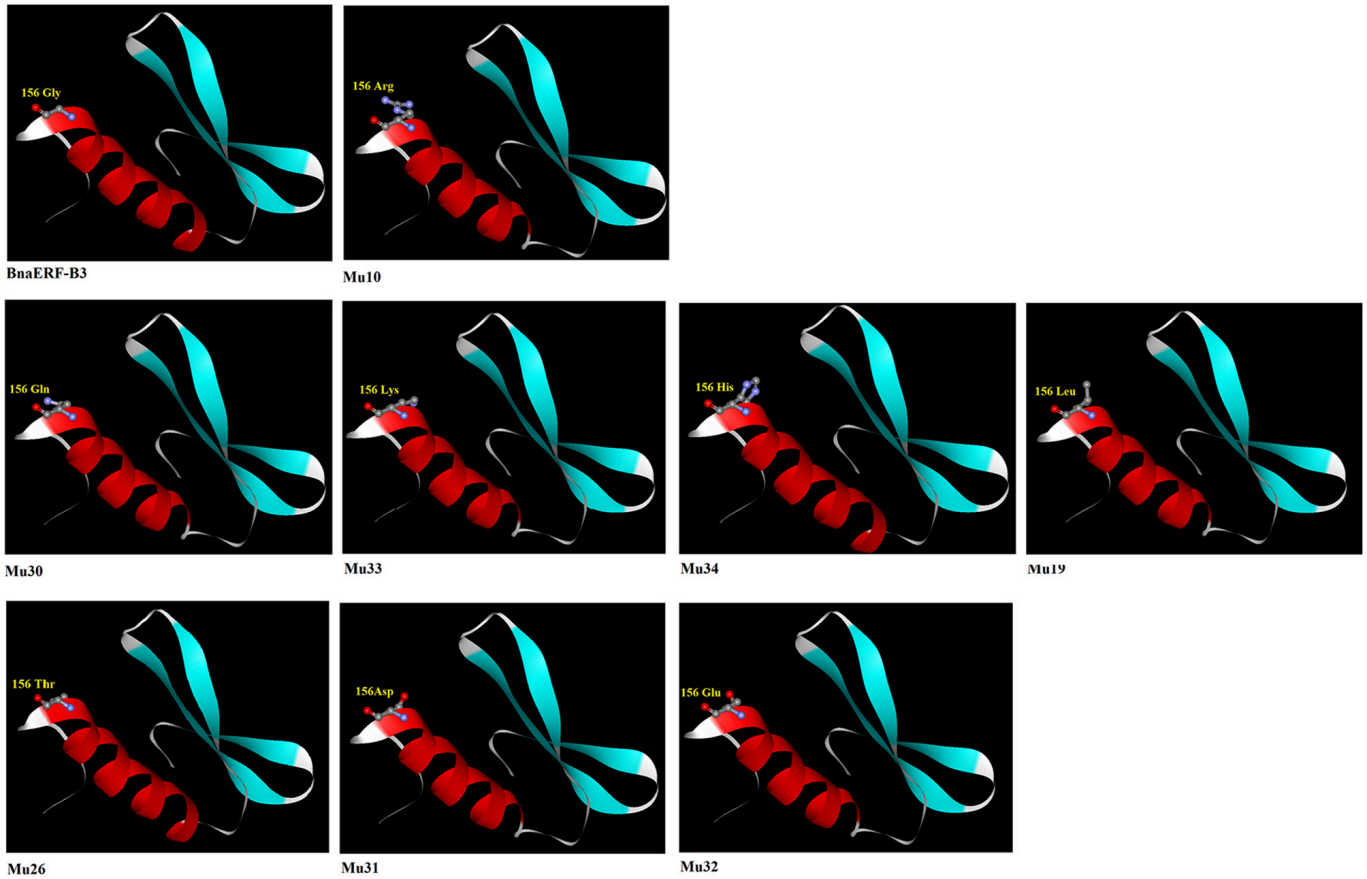
**The molecular modeling of the conserved AP2 domain among the BnaERF-B3 and mutants**. The different residues are indicated as ball and stick representation. Red ball represents O atom, blue ball represents N atom, and gray ball represents C atom.

Numerous studies have proved that the structure of amino acids considerably affects the protein structure and conformation. Studies on ERF proteins also showed that sequence diversity in the ERF domain can indirectly affect the binding activity to the GCC box (Ohta et al., 2001; Jin et al., 2010a). Among the 12 amino acid site substitutions, only Gly156Arg is located in the AP2-domain. The mutant hosting Gly156Arg (Mu10) showed the highest binding activity to the GCC box, followed by Gly156Gln (Mu30), Gly156Lys (Mu33), Gly156His (Mu34), and Gly156Leu (Mu19). However, the mutants Gly156Thr (Mu26), Gly156Asp (Mu31), and Gly156Glu (Mu32) showed no detectable binding activity to the GCC box. The 3D structures of the mutant and wild-type BnaERF-B3 were derived using the Swiss–Model server, with AtERF1 as the template (Figure 6). Comparison of the mutated amino acid residue sequences and the structure of BnaERF-B3 indicated that the 3D structures have no visible differences in the three β-sheets. However, an obvious change in the α-helix structure occurred when the amino acid residues were substituted. In the mutants which had high binding activity, more N atom (blue ball) were be found in models. The different amino acids in site 156th represented different conformation, which may lead to activity change.

### Identification of key sites in AP2-domain for binding activity to the GCC box

Previous studies have showed that some residues in AP2-domain of ERFs were indispensable for binding with GCC box (Jofuku et al., 1994; Allen et al., 1998; Cao et al., 2001; Hao et al., 2002; Liu et al., 2006). Sequence alignment revealed that these residues were not found on the BnaERF-B3. The mutant Mu3 of BnaERF-B3 showed highest binding activity to GCC box, it is reasonable to deduce that Arg156 is responsible for the binding activity of GCC box. To further validate the residues in the AP2-domain regions that might be responsible for the activity of ERFs to activate transcription, a series of site-directed mutants (Mu35–Mu42) were further constructed according to the different sites of sequence alignment of Mu3 and other ERFs, which reported by other researchers (Jofuku et al., 1994; Allen et al., 1998; Cao et al., 2001; Hao et al., 2002; Liu et al., 2006). All the mutants were transformed into yeast cells and the interaction with GCC box were quantified with X-gal assay.

**Figure 7:**
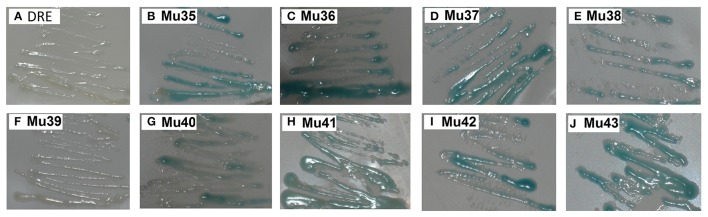
**Yeast one-hybrid analysis of activities of mutants of and Mu35 to Mu43**. The yeast cells were examined with X-gal after growing on SD/-Ura /-Trp- medium for 2 d at 30°C. DRE: yeast reporter cells carry DRE element (as a control). The information of the substitution site and amino acid of all Mu35 to Mu42 were as follows: Mu35: Pro115Asn; Mu36: Trp116Ser; Mu37: Ala120Val; Mu38: Asp125Glu; Mu39: Gly136Glu; Mu40: Gly136Ser; Mu41: Ser140Thr; Mu42: Ala144Val. Mu43 contained all the mutation sites of Mu35 to Mu42.

**Figure 8:**
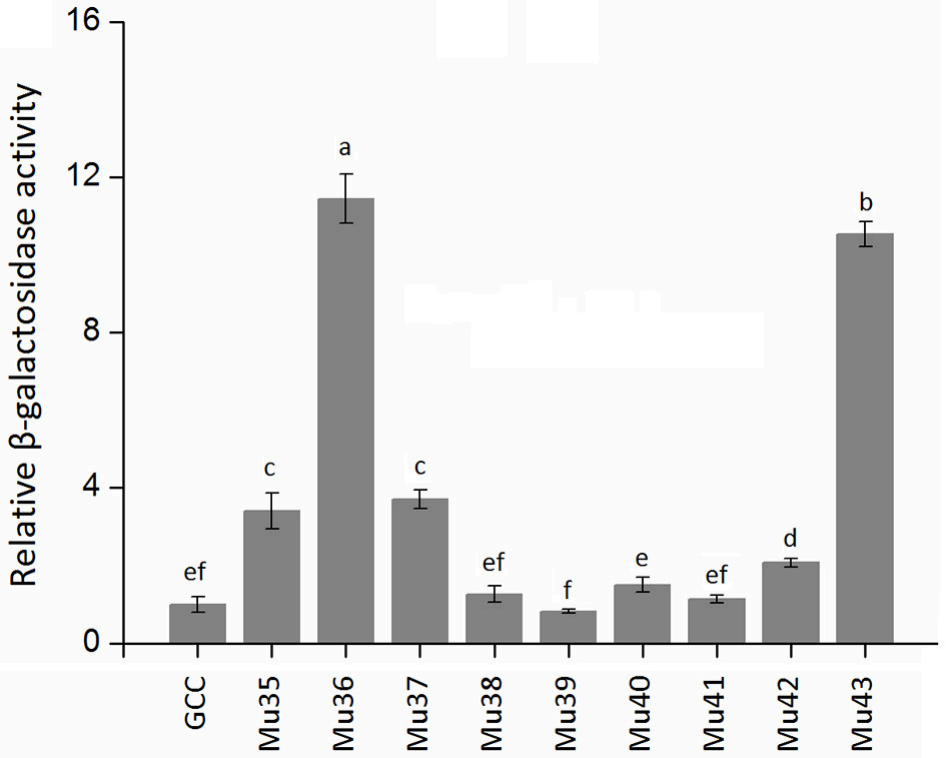
**The relative β-galactosidase activities of Mu35 to Mu43**. The data are the means ± SD of three replicates. Different letters on the vertical bars indicate significant difference at 0.05 levels.

Figure 7 showed that the mutants still showed weak or strong binding activity to GCC box, except Mu39. The synthetic mutant Mu43, which contained all the mutation sites of Mu35 to Mu42, exhibited a higher binding activity. On the basis of Gly156 was replaced by Arg, the other amino acid sites have little effect on the binding with GCC box. β-galactosidase activity assay also showed a consistent result (Figure 8). Mu36 (Trp116Ser) had the highest binding activity, followed by Mu43, Mu35 (Pro115Asn). In tobacco, the substitution of Pro9 or Trp10 with Asn or Ser in AP2-domain led to increase of the binding activity of NtERF2-F (Hao et al., 2002). Together, these results strongly suggested that the binding specificity with *cis* element can be altered by amino acid exchange mutation, and also demonstrated that Arg156 is an important determinant of the Bna-ERF-B3 binding specificity.

### Identification of gly in AP2-domain of DREBs for binding activity to the DRE *cis* element

**Figure 9:**
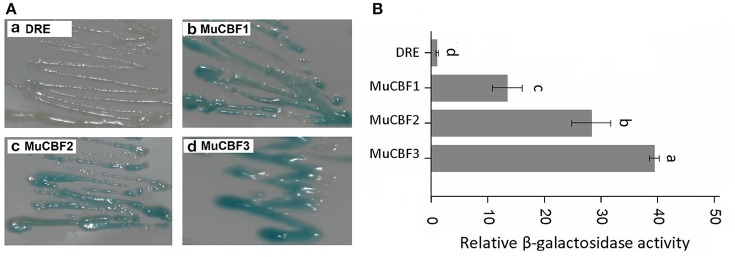
**Yeast one-hybrid analysis of MuCBF1, MuCBF2, and MuCBF3. (A)** The binding activities of MuCBF1, MuCBF2, and MuCBF3. **(B)** The relative β-galactosidase activities of MuCBF1, MuCBF2, and MuCBF3. DRE: yeast reporter cells carry DRE element (as a control). The data are the means ± SD of three replicates. Different letters on the vertical bars indicate significant difference at 0.05 levels.

As the members of AP2/ERF family, ERFs and DREBs have high similarity on amino acid sequences. Cao et al. (2001) and Hao et al. (2002) have reported that Val14 and Glu19 residues were crucial in the regulation of the binding activity of DREB1A to the DRE *cis* element, while Ala14 was an important determinant of the ERF binding specificity. Three DREB proteins (CBF1, CBF2, and CBF3) in *Arabidopsis* have been identified to bind with DRE element (Liu et al., 1998). Amino acid sequence analysis of BnaERF-B3 and these three DREB proteins indicated that the AP2-domains were also highly homologous. We found that in the AP2-domains of CBFs of *Arabidopsis*, the corresponding amino acids of Gly156 in BnaERF-B3 were all Arg. To explore whether Gly can affect the binding activity of DREB proteins, three mutants MuCBF1 (Arg95Gly), MuCBF2 (Arg98Gly), and MuCBF3 (Arg98Gly) of *Arabidopsis* were obtained. Yeast one-hybrid (Figure 9A) and β-galactosidase activity (Figure 9B) showed that these three mutants also had the strong binding ability with DRE element. This result showed Gly did not affect the binding affinity of DREBs to DRE *cis* element.

## Discussion

Amino acid sequences can determine the higher-order of protein structure when the specific conformation of the protein exhibits biological function. A single amino acid mutation may cause functional disorder in a protein. Sickle cell anemia is a disease caused by the replacement of a Glu acid residue with Val at the sixth position of the β-chain of hemoglobin (Mehanna, 2001). A study on the recombinant encephalomyocarditis (EMC) virus showed that a point mutation at position 776 (Ala776Thr) of the EMC virus polyprotein resulted in a loss of viral diabetogenicity (Jun et al., 1997). The modification of peptides and proteins has attracted wide spread attention and has become an important research direction; several enzymes and proteins have been successfully modified to increase their efficiency, investigate gene functions, and realize molecular breeding (Lassner and Bedbrook, 2001; Eijsink et al., 2005; Xiong et al., 2007a, 2011; Reinstädler et al., 2010). Common strategies include random-priming *in vitro* recombination, gene shuffling, site-directed mutagenesis, and semi-rational design (Stemmer, 1994; Xiong et al., 2007b,c, 2010; Tian et al., 2013, 2015). Site-directed mutagenesis is widely used in research on proteins to reveal the relationship between structure and function. Several studies have been performed to explore key amino acid residues. For example, when residue Ala363 was changed to Val, the cytochrome CYP2B2 mutant protein exhibited an altered metabolite profile; by contrast, substitution of Ala478 with Gly yielded a higher catalytic efficiency for lidocaine oxidation than that of CYB2B2 (Domanski and Halpert, 2001). A mutation at Glu160 in the active site of trichosanthin caused a significant loss of potency, whereas substitution of Gln156 with Ala only slightly decreased the activity (Wong et al., 1994).

Transcription factors can directly regulate downstream genes by specifically binding *cis*-acting elements in the promoter of target genes at their DNA-binding site. Various studies have confirmed that transcription factors play pivotal roles in many processes during plant growth, such as integration of various signal transduction pathways and responses to abiotic/biotic stresses (Kasuga et al., 1999; Yamaguchi-Shinozaki and Shinozaki, 2006; Nakashima et al., 2012). ERF transcription factors were shown to be excellent candidates for improving multiple stress resistance by regulating the expression of various stress-inducible genes (Gutterson and Reuber, 2004; Xu et al., 2011; Mizoi et al., 2012; Rong et al., 2014). The special DNA-binding domains of ERFs have been well characterized, which mainly recognize the *cis*-acting element AGCCGCC, known as the GCC box (Ohme-Takagi and Shinshi, 1995; Hao et al., 1998; Zhang G. et al., 2009).

Our previous work found that an ERF member named BnaERF-B3 could not activate the expression of the reporter gene in yeast, whereas the mutants of BnaERF-B3 (Mu1, Mu2, and Mu3) showed binding activity with GCC box (Jin et al., 2010a). Overexpressing *BnaERF-B3-mu3* exhibited more freezing tolerance than the original *BnaERF-B3* in transgenic *Arabidopsis* (Xiong et al., 2013). To identify the key amino acids that are crucial to the binding activity of BnaERF-B3, site-directed mutagenesis was used to generate a series of mutants.

We first produced 11 BnaERF-B3 mutants containing mutant amino acid residues. The relative activity of Mu10 was higher than that of BnaERF-B3. The results showed that the mutation in the amino acid Arg156 was a potential key site involved in binding activity. The mutations with other amino acids outside the AP2-domain were not essential for binding activity. The key amino acid Arg156 was located in the α-helix. Although ERF transcription factor members present high similarity in the conserved AP2-domain, the differences of amino acid residues in their domains lead to ERFs with different levels of binding activity to the GCC box. The conserved Ala37 in the α-helix was confirmed to be essential for binding to the GCC box (Liu et al., 2006). Thr178 was participated in forming α-helix structures (Allen et al., 1998). Other studies also strongly suggest that the α-helix was important for AP2-domain function in ERF proteins and may participate in protein–protein interactions (Jofuku et al., 1994; Büttner and Singh, 1997).

Besides Arg156, when Gly156 was substituted with other four amino acids (Gln, Lys, His, and Leu), the proteins also showed high binding activity. However, proteins hosting other three amino acids (Thr, Asp, and Glu) showed no detectable binding activity. As compared with Gly, the other aliphatic amino acids (Leu, Val, and Ile) seemed improve the affinity of ERF factor with GCC box, especially Leu. Here, we also found that the introduction of basic amino acid residues (Arg, Gln, Lys, and His) caused the increasing of binding activity, while replacements with acidic amino acid residues (Asp and Glu) appeared to have less impact. This result showed that acidic amino acid residues might interfere with the DNA binding while other types of amino acids seem to be compatible or advantageous. Previous studies also found that in some experiments acidic amino acid-rich amino-terminus did not have nucleic acid binding activity (Frédéric et al., 2011). The residues in AP2-domain of ERF genes were indispensable for binding with GCC box. Multiple sequence analysis results showed that several amino acids, such as Asp43 and Asn57, are highly conserved in the AP2-domain of ERF proteins (Hao et al., 1998; Nakano et al., 2006). Gly174 was necessary for AP2 genetic functions; the replacement of Gly residues with either Ser or Glu may perturb its function (Jofuku et al., 1994). Two mutations of NtERF2-F, ERFpn and ERFws, exhibited a higher binding activity with GCC box (Hao et al., 2002). A series of mutations was further performed to explore the optimal amino acid in AP2-domain for BnaERF-B3 binding to the GCC box. Analysis of the result revealed that the amino acid residues of AP2-domain are sufficient to affect the binding activity of ERF to GCC box. Especially Pro115Asn and Trp116Ser can obviously increase the binding activity. The change in the protein activity may be due to the different amino acids that cause a conformational change in the protein.

The amino acid residues of AP2-domains are also conserved in DREB subgroup as well as ERF subgroup. To assess the functional significance of the corresponding residue of ERF subgroup in binding to DRE element, three mutants MuCBF1 (Arg95Gly), MuCBF2 (Arg98Gly), and MuCBF3 (Arg98Gly) were obtained. The result revealed that the substitution of Arg with Gly did not affect the binding activity of the DREB transcription factors to the DRE *cis* element. The findings in this study can be used for further research on AP2/ERF protein functions and plant molecular breeding in the future.

## Author contributions

AX initiated and designed the research. JZ, ML, BW, YL, and AX performed the experiments. JZ, ML, and AX analyzed the data. AX contributed reagents/materials/analysis tools. JZ and ML wrote the paper. JZ, ML, and AX revised the paper.

### Conflict of interest statement

The authors declare that the research was conducted in the absence of any commercial or financial relationships that could be construed as a potential conflict of interest.

## Supplementary material

Figure S1 **Alignment of amino acid sequences in AP2-domain of BnaERF-B3 and the mutants**. The numbers above the amino acids corresponding to the position of the difference amino acid between BnaERF-B3 and the mutants. The α-helix and β-sheet regions are marked with black bar and arrows, respectively.

Table S1 **Primers used for cloning or synthesizing mutations**. F, Forward primer; R, Reverse primer. – represents the site was lacking. Due to the mutation site of Mu13, Mu14, Mu15, and Mu16 was near to the termination, so we only designed the reverse primer.
