# Supplementary figures and images for: Arg156 in the AP2-Domain Exhibits the Highest Binding Activity among the 20 Individuals to the GCC Box in BnaERF-B3-hy15, a Mutant ERF Transcription Factor from Brassica napus
Source: Front Plant Sci. 2016 Oct 27;7:1603. doi: 10.3389/fpls.2016.01603 (PMC5081391; doi:10.3389/fpls.2016.01603)

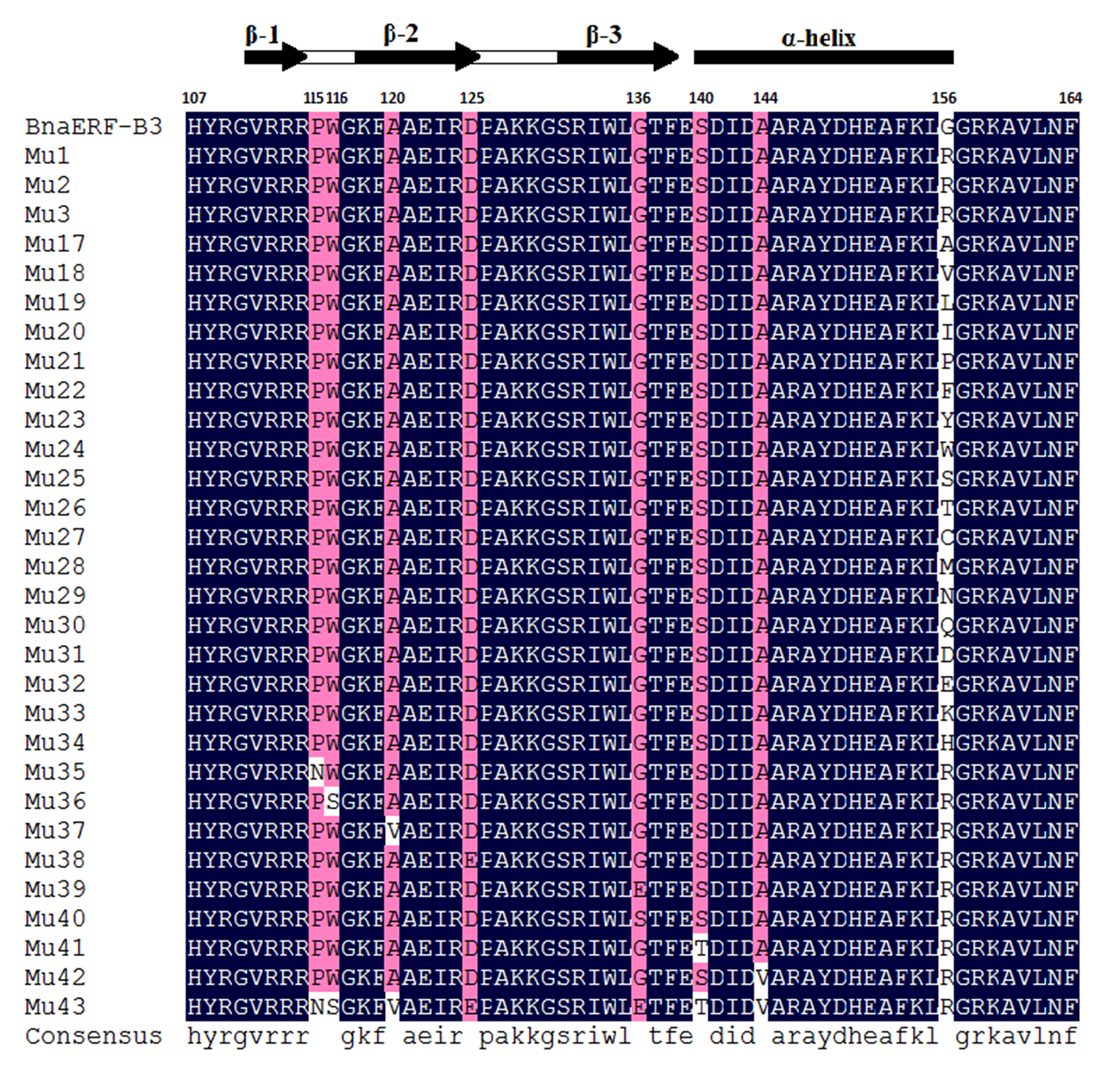

Supplement: Figure S1 — Alignment of amino acid sequences in AP2-domain of BnaERF-B3 and the mutants. The numbers above the amino acids corresponding to the position of the difference amino acid between BnaERF-B3 and the mutants. The α-helix and β-sheet regions are marked with black bar and arrows, respectively. [file Image1.TIF]
