# Supplementary material for: Arg156 in the AP2-Domain Exhibits the Highest Binding Activity among the 20 Individuals to the GCC Box in BnaERF-B3-hy15, a Mutant ERF Transcription Factor from Brassica napus
Source: Front Plant Sci. 2016 Oct 27;7:1603. doi: 10.3389/fpls.2016.01603 (PMC5081391; doi:10.3389/fpls.2016.01603)
Supplement: Table S1 — Primers used for cloning or synthesizing mutations. F, Forward primer; R, Reverse primer. – represents the site was lacking. Due to the mutation site of Mu13, Mu14, Mu15, and Mu16 was near to the termination, so we only designed the reverse primer. [file DataSheet1.docx]

**Table S1** Primers used for cloning or synthesizing mutations.

F: Forward primer; R: Reverse primer. -- represents the site was lacking.

Note: Due to the mutation site of Mu13, Mu14, Mu15 and Mu16 was near to the termination, so we only designed the reverse primer.

| Mutation | Mutate form | Primer’s name | Primer sequence 5’-3’ |
| --- | --- | --- | --- |
| BnaERF-B3 | For cloning | BnaERF-B3-F | GGATCCATGGCAACTATTGAGGAAATCTCTG |
|  |  | BnaERF-B3-R | GAGCTCTCAGTTTGATGATGAATTGCTCG |
| Mu4 | Met19Leu | Mu4-F | TGAAGACTTGTTGATCCCTGATGG |
|  |  | Mu4-R | TCAGGGATCAACAAGTCTTCAAAG |
| Mu5 | Gln51Leu | Mu5-F | TTCCTAAACTGGAGCCTAGTTCAC |
|  |  | Mu5-R | ACTAGGCTCCAGTTTAGGAACTTG |
| Mu6 | Ala57Val | Mu6-F | CTAGTTCACCTGTTCTTGATCCAG |
|  |  | Mu6-R | GATCAAGAACAGGTGAACTAGGCT |
| Mu7 | Phe62Ser | Mu7-F | GATCCAGATTCGTATGTCCAAGAG |
|  |  | Mu7-R | TTGGACATACGAATCTGGATCAAG |
| Mu8 | Met70Thr | Mu8-F | TCTGCAAACAGAAGCAGAATCATC |
|  |  | Mu8-R | TTCTGCTTCTGTTTGCAGAAACTC |
| Mu9 | Thr78--,  Thr79-- | Mu9-F | AACAACAACAACAACTACAACATC |
|  |  | Mu9-R | TTGTAGTTGTTGTTGTTGTTGATG |
| Mu10 | Gly156Arg | Mu10-F | CTTTTAAGCTCAGGGGAAGAAAAG |
|  |  | Mu10-R | TCTTCCCCTGAGCTTAAAAGCTTC |
| Mu11 | Tyr171Cys | Mu11-F | GCAGGAAAGTGTGATGCTCCGGTC |
|  |  | Mu11-R | CGGAGCATCATACTTTCCTGCGTC |
| Mu12 | Asn184Cys | Mu12-F | GAGGAGAAGATGCGATGTGCCGGA |
|  |  | Mu12-R | GGCACATCGCATCTTCTCCTCTTC |
| Mu13 | Thr192Val,  Thr193Ala | Mu13-R | GAGCTCGTTTGATGATGAATTGCTCGATGCTATTCCTTGA |
| Mu14 | Thr192Ile,  Thr193Ala | Mu14-R | GAGCTCGTTTGATGATGAATTGCTCGTAGCTACTCCTTGA |
| Mu15 | Gly156Arg,  Thr192Val | Mu15-R | gagctctcagtttgatgatgaattgctcgtagttactccttgagg |
| Mu16 | Gly156Arg,  Thr192Ile | Mu16-R | gagctctcagtttgatgatgaattgctcgtagttattccttgagg |
| Mu17 | Gly156Ala | Mu17-F | cttttaagctcgCgggaagaaaag |
|  |  | Mu17-R | TCTTCCCGCGAGCTTAAAAGCTTC |
| Mu18 | Gly156Val | Mu18-F | cttttaagctcgTgggaagaaaag |
|  |  | Mu18-R | TCTTCCCACGAGCTTAAAAGCTCC |
| Mu19 | Gly156Leu | Mu19-F | cttttaagctcCTAggaagaaaag |
|  |  | Mu19-R | TCTTCCTAGGAGCTTAAAAGCTTC |
| Mu20 | Gly156Ile | Mu20-F | cttttaagctcATTggaagaaaag |
|  |  | Mu20-R | TCTTCCAATGAGCTTAAAAGCTTC |
| Mu21 | Gly156Pro | Mu21-F | cttttaagctcCCgggaagaaaag |
|  |  | Mu21-R | TCTTCCCGGGAGCTTAAAAGCTTC |
| Mu22 | Gly156Phe | Mu22-F | cttttaagctcTTTggaagaaaag |
|  |  | Mu22-R | TCTTCCAAAGAGCTTAAAAGCTTC |
| Mu23 | Gly156Tyr | Mu23-F | cttttaagctcTATggaagaaaag |
|  |  | Mu23-R | TCTTCCATAGAGCTTAAAAGCTTC |
| Mu24 | Gly156Trp | Mu24-F | CttttaagctcTggggaagaaaag |
|  |  | Mu24-R | TCTTCCCCAGAGCTTAAAAGCTTC |
| Mu25 | Gly156Ser | Mu25-F | cttttaagctcTCAggaagaaaag |
|  |  | Mu25-R | TCTTCCTGAGAGCTTAAAAGCTTC |
| Mu26 | Gly156Thr | Mu26-F | cttttaagctcACAggaagaaaag |
|  |  | Mu26-R | TCTTCCTGTGAGCTTAAAAGCTTC |
| Mu27 | Gly156Cys | Mu27-F | cttttaagctcTGCggaagaaaag |
|  |  | Mu27-R | TCTTCCGCAGAGCTTAAAAGCTTC |
| Mu28 | Gly156Met | Mu28-F | cttttaagctcATgggaagaaaag |
|  |  | Mu28-R | TCTTCCCATGAGCTTAAAAGCTTC |
| Mu29 | Gly156Asn | Mu29-F | cttttaagctcAACggaagaaaag |
|  |  | Mu29-R | TCTTCCGTTGAGCTTAAAAGCTTC |
| Mu30 | Gly156Gln | Mu30-F | cttttaagctcCAAggaagaaaag |
|  |  | Mu30-R | TCTTCCTTGGAGCTTAAAAGCTTC |
| Mu31 | Gly156Asp | Mu31-F | cttttaagctcGATggaagaaaa |
|  |  | Mu31-R | TCTTCCATCGAGCTTAAAAGCTTC |
| Mu32 | Gly156Glu | Mu32-F | cttttaagctcgAAggaagaaaag |
|  |  | Mu32-R | TCTTCCTTCGAGCTTAAAAGCTTC |
| Mu33 | Gly156Lys | Mu33-F | cttttaagctcAAgggaagaaaag |
|  |  | Mu33-R | TCTTCCCTTGAGCTTAAAAGCTTC |
| Mu34 | Gly156His | Mu34-F | CTTTTAAGCTCCATGGAAGAAAAG |
|  |  | Mu34-R | TCTTCCATGGAGCTTAAAAGCTTC |
| Mu35 | Pro115Asn | Mu35-F | AGGAGGAACTGGGGCAAATTCGCA |
|  |  | Mu35-R | GAATTTGCCCCAGTTCCTCCTTCT |
| Mu36 | Trp116Ser | Mu36-F | AGGAGGCCAAGCGGCAAATTCGCA |
|  |  | Mu36-R | GAATTTGCCGCTTGGCCTCCTTCT |
| Mu37 | Ala120Val | Mu37-F | TTCGTAGCAGAGATTCGAGATCCG |
|  |  | Mu37-R | ATCTCGAATCTCTGCTACGAATTT |
| Mu38 | Asp125Glu | Mu38-F | GATTCGAGAGCCGGCTAAGAAAGG |
|  |  | Mu38-R | TTCTTAGCCGGCTCTCGAATCTC |
| Mu39 | Gly136Glu | Mu39-F | ATTTGGCTAGAGACTTTTGAGAGT |
|  |  | Mu39-R | CTCAAAAGTCTCTAGCCAAATCCT |
| Mu40 | Gly136Ser | Mu40-F | ATTTGGCTAAGCACTTTTGAGAGT |
|  |  | Mu40-R | CTCAAAAGTGCTTAGCCAAATCCT |
| Mu41 | Ser140Thr | Mu41-F | TTTGAGACTGATATTGATGCTGCA |
|  |  | Mu41-R | AGCATCAATATCAGTCTCAAAAGT |
| Mu42 | Ala144Val | Mu42-F | ATTGATGTTGCAAGAGCTTATGAC |
|  |  | Mu42-R | ATAAGCTCTTGCAACATCAATATC |
| MuCBF1 | Arg95Gly | MuCBF1-F | GCCCTCGGTGGCCGATCAGCATGT |
|  |  | MuCBF1-R | TGCTGATCGGCCACCGAGGGCTAA |
| MuCBF2 | Arg98Gly | MuCBF2-F | GCTCTCGGTGGCAGATCTGCCTGT |
|  |  | MuCBF2-R | GGCAGATCTGCCACCGAGAGCTAT |
| MuCBF3 | Arg98Gly | MuCBF3-F | TTAGCCCTTGGTGGCCGATCAGCC |
|  |  | MuCBF3-R | TGATCGGCCACCAAGGGCTAAAGC |
| CBF1 | For cloning | CBF1-F | ATGAACTCATTTTCAGCTTTTTCTG |
|  |  | CBF1-R | TTAGTAACTCCAAAGCGACACG |
| CBF2 | For cloning | CBF2-F | ATGAACTCATTTTCTGCCTTTTCT |
|  |  | CBF2-R | TAATAGCTCCATAAGGACACGT |
| CBF3 | For cloning | CBF3-F | ATGAACTCATTTTCTGCTTTTTCTG |
|  |  | CBF3-R | TTAATAACTCCATAACGATACGTCG |
